# The impact of the US president’s emergency plan for AIDS relief (PEPFAR) HIV and AIDS program on the Nigerian health system

**DOI:** 10.11604/pamj.2016.25.143.9987

**Published:** 2016-11-11

**Authors:** Florence Femi Odekunle, Raphael Oluseun Odekunle

**Affiliations:** 1 Queen Margaret University, Institute for Global Health and Development, Edinburgh, UK; 2 University of Ilorin, Department of Epidemiology and Community Health, Kwara State, Nigeria

**Keywords:** HIV/AIDS program, PEPFAR, impact, Nigeria, health system

## Abstract

The PEPFAR HIV/AIDS program has had noticeable impacts on the Nigerian health system. The impacts are presented using the World Health Organization (WHO) health system six building blocks. These include service delivery, health workforce, health information, medical products, vaccines and technologies, financing and governance. PEPFAR HIV/AIDS program has improved the delivery of prevention and care services for people living with HIV/AIDS (PLWHA). The most important measure of PEPFAR’s success is the availability of free ART in Nigeria for PLWHA who need this. The PEPFAR program has brought about increased political awareness of and raised the priority given to public health by governments and civil society through its scaling up response to HIV/AIDS. The scaled-up program has direct benefits on the health workforce by preserving HIV-infected health personnel’s lives so that they can live longer enjoy a better quality of life and return to their jobs; all of which invariably enhances the country’s health workforce. Moreover, the training and retraining in PEPFAR HIV/AIDS program have boosted both the morale and the skills of the health workforce. Considerable resources have been brought into Nigeria for scaled-up HIV/AIDS treatment by PEPFAR. However, this has contributed to the development of donor dependency syndrome by Nigerian government. There is a non-alignment between PEPFAR HIV/AIDS program and the recipient country’s health system. Attention to maternal mortality and other reproductive health services has suffered as non-governmental organizations (NGOs) pursue AIDS money and local governments receive signals from the political center to prioritize HIV/AIDS over other problems that are just as serious. A functional health system is important in prevention of the HIV epidemic. Hence efforts should be made to strengthen health systems. The PEPFAR HIV/AIDS program should be harmonized with the country’s health system for maximum impact.

## Introduction

The emergence of Human Immunodeficiency Virus (HIV) in the past three decades has presented the most severe challenge to governments, the health workforce and society at large. HIV/AIDS is regarded as one of the major health crises of the twenty first century. The severity of the epidemic has led to implementation of various interventions in different parts of the world, especially in the most affected regions, all aiming to reduce its spread and save the lives of already infected people. In light of this, PEPFAR has been actively engaged in the fight against HIV/AIDS through provision of free antiretroviral therapy (ART) for people living with HIV/AIDS (PLWHA) who need this. In order to understand the impact of the PEPFAR HIV program on the Nigerian health system, this paper utilizes the WHO health system framework.

## Seminar

### The WHO health system framework overview

According to the WHO, “a health system comprises all the organizations, institutions and resources that are devoted to producing health actions” [1]. The WHO Health system framework is a single framework with six building blocks. The main aim of this framework is to give a clear understanding of what a health system is and what constitutes health systems strengthening. It defines a distinct number of ‘building blocks’ these include service delivery, health workforce, Information, medical products, vaccines and technologies, financing and leadership/governance [1].

### Background information on HIVAIDS in Nigeria

Nigeria is the most populous African country with a population of about 152 million in 2010 [2]. Alubo reports that the first AIDS case in Nigeria was diagnosed in 1986 in a 13-year-old female street hawker [3]. The prevalence rate of the disease in 1991 was 1.8 percent, but this increased rapidly to about 5.1 percent in 2001 [2]. According to the 2010 USAID report, 2,980,000 people are living with HIV/AIDS in Nigeria [2]. At first the government was slow to respond to the increasing rates of HIV transmission because of widespread denial by the political leaders, especially during the military regime, and also by many family members [3]. For many, AIDS was either denied as unreal or seen as too distant to arouse any anxiety [3] and it was only in 1991 that the federal ministry of health made their first attempt to assess Nigeria’s AIDS situation [3]. Perhaps because of this thick wall of silence, Nigeria is not included among the recognized areas of high HIV/AIDS prevalence in Africa, or the so-called AIDS belt [3]. The Nigerian government adopted the National policy on HIV/AIDS control program in 1997 with the aim of countering the devastating effects of the disease on social and economic development.

### PEPFAR BACKGROUND INFORMATION

PEPFAR is one of the global health initiatives (GHIs) that was established in 2003 by U.S. President George W. Bush in response to the HIV/AIDS epidemics. PEPFAR is centrally managed by the Office of the US Global AIDs Coordinator (OGAC) in conjunction with other United States Government (USG) agencies [4]. In Nigeria, the PEPFAR initiative is coordinated by three USG agencies, namely: USAID, the Centers for Disease Control and prevention (CDC), and the Defense Department [2]. Huge resources have been brought into countries including Nigeria for HIV/AIDS programs organized by PEPFAR The PEPFAR general policy guidance for all bilateral programs includes adherence to emergency plan policy: all USG bilateral programs receiving resource for HIV/AIDS regardless of programs size or funding account source, are expected to follow the policies of PEPFAR such as ABC guidance (abstinence, be faithful, and condom use) [**5**].

### Impact of the PEPFAR HIV/AIDS program on the Nigerian health system

The PEPFAR HIV/AIDS program has had noticeable impacts on the Nigerian health system. Here, both its positive and negative impacts will be presented using the WHO health system building blocks.

### Service delivery

USAID highlights that the most important measure of PEPFAR’s success is the availability of free ART in Nigeria for PLWHA who need this [**2**]. PEPFAR HIV/AIDS program has improved the delivery of prevention and care services for PLWHA [**6**]. Many lives have been saved through this treatment. The number of PLWHA receiving HAART (highly active antiretroviral therapy) has gone up [**7**]. Baker states that the treatment has freed up hospital bed spaces which can be used for other purposes [**6**]. Moreover, the scaled-up program offers free counseling and laboratory services to people who do not yet know their HIV status and there are also laboratory monitoring services for PLWHA who are receiving treatment to monitor their improvement [**2**]. In addition, two years ago the PEPFAR HIVAIDS program included some preventive measures, such as those to prevent mother-to-child transmission of HIV (PMTCT) [**2**].

### Governance/ Leadership

According to Dongbao et al. “the centrality to all national health systems is the need for effective governance” [8]. The performance of the health system is dependent on the overall governance of a country [9]. The PEPFAR program has brought about increased political awareness of and raised the priority given to public health by governments and civil society through its scaling up response to HIV/AIDS [8]. Similarly, there have been changes in health policies. For instance, before the advent of the PEPFAR HIV/AIDS program, the policy of user fees for all aspects of HIV/AIDS care, including tests remained, but with PEPFAR it was changed to a free ART policy [10]. Furthermore, there is greater stakeholder participation and channeling of funds to non-governmental stakeholders and faith-based bodies such as the Christian Health Association of Nigeria [4]. Dongbao and colleagues state that “AIDS treatment activism has promoted access to basic medicines, including ARV drugs for the underserved, especially women, and has reduced health care inequities” [8]. HERFON notes that females access ART more than men in Nigeria, and this is similar to findings in other Sub-Saharan Africa countries [11]. This is because females use health facilities more than males. The ratio is three to two. However, Biesma and others claims that the program has caused a distraction from having coordinated efforts to strengthen health systems because it takes a vertical approach to planning, management, monitoring and evaluation systems [12]. There is a non-alignment between PEPFAR HIV/AIDS program and the recipient country’s health system [12]. Attention to maternal mortality and other reproductive health services has suffered as NGOs pursue AIDS money and local governments receive signals from the political center to prioritize HIV/AIDS over other problems that are just as serious [13]. The planning activities of the PEPFAR HIV/AIDS initiative remain top-down. It does not draw on stakeholders’ knowledge in programme development before designing. Funds are disbursed directly from Washington DC, through existing US agencies, to the country [14]. There is little policy discussion on strategy at the country level as all the directives on how to operate are designed by the US [14]. The power increasingly exercised by PEPFAR carries implications for the national government [14]. For instance, the imposition of the donor policy of ABC: abstinence, be faithful to your partner and condom use. Condoms are to be provided and promoted for only high risk behaviors and persons such as prostitutes and IDU (intravenous drug user). PEPFAR’s prevention approach of over emphasizing the AB only and discouraging condom promotion has been shunned by many activists, likewise the roles of faith-based organizations in a multi-religious setting, which in many ways determine its own moral values and impose them on recipient countries [15].

### Health workforce

The aim in a workforce is to get the right workers, with the right skills, in the right place doing the right things [16]. The scaled-up program has direct benefits on the health workforce by preserving HIV-infected health personnel’s lives so that they can live longer enjoy a better quality of life and return to their jobs; all of which invariably enhances the country’s health workforce [6]. Moreover, the training and retraining in PEPFAR HIV/AIDS program have boosted both the morale and the skills of the health workforce [11]. It has also been argued that the HIV/AIDS initiative has caused some setback in health workforce. This is because the program is more attractive to and lucrative for many public health workers than other sectors of the healthcare, so they have moved into the HIV/AIDS program, which has resulted in insufficient personnel in other areas [11]. In addition, a brain-drain of public health providers to well-funded HIV-related NGOs as a result of the scaled-up ART program has been reported [8].

### Financing

USAID clearly states that considerable resources have been brought into Nigeria for scaled-up HIV/AIDS treatment by PEPFAR [**2**]. For instance, in fiscal year 2009, Nigeria received four hundred and thirty-eight million US dollars from PEPFAR. However, AVERT notes that this has contributed to the development of donor dependency syndrome by Nigerian government and this is often reflected in its financial contribution which is only five percent of the funds for the ART program [10].

### Information

In Nigeria sharing of information among government and civil society organizations has greatly improved because of the scaled-up program [11]. In addition, information on health is easily and readily available. Conversely, the PEPFAR HIV/AIDS program overlaps with the Global Fund Initiative in Nigeria, which has resulted in a burden of duplicative reporting forms from multiple programs [17]. It has been noted that many GHIs do not wish to be coordinated because of cost and loss of autonomy entailed and this has resulted in priority duplication and fragmentation of services [18].

### Medical products and technologies

Efficient drug procurement and supply systems are very important in health systems in order to achieve equal access to essential drugs [19]. The PEPFAR HIV program in Nigeria has brought significant improvement in both logistics and supply systems of ART and other laboratory materials. PEPFAR, however, uses only patented medications, so a lot of money goes on medicines.

## Conclusion

The PEPFAR HIV/AIDS program has had noticeable impacts on the Nigerian health system. The impacts are presented using the WHO health system six building blocks. These include service delivery, health workforce, health information, medical products, vaccines and technologies, financing and governance. PEPFAR HIV/AIDS program has improved delivery of prevention and care services for people living with HIV/AIDS (PLWHA). The most important measure of PEPFAR’s success is the availability of free ART in Nigeria for PLWHA who need this. The PEPFAR program has brought about increased political awareness of and raised the priority given to public health by governments and civil society through its scaling up response to HIV/AIDS. The scaled-up program has direct benefits on the health workforce by preserving HIV-infected health personnel’s lives so that they can live longer enjoy better quality of life and return to their jobs; all of which invariably enhances the country’s health workforce. Moreover, the training and retraining in PEPFAR HIV/AIDS program have boosted both the morale and the skills of health workforce. Considerable resources have been brought into Nigeria for scaled-up HIV/AIDS treatment by PEPFAR. However, this has contributed to the development of donor dependency syndrome by Nigerian government. There is a non-alignment between PEPFAR HIV/AIDS program and the recipient country’s health system. Attention to maternal mortality and other reproductive health services has suffered as NGOs pursue AIDS money and local governments receive signals from the political center to prioritize HIV/AIDS over other problems that are just as serious. A functional health system is important in prevention of the HIV epidemic. Hence efforts should be made to strengthen health systems. The PEPFAR HIV/AIDS program should be harmonized with the country’s health system for maximum impact. The WHO in conjunction with PEPFAR and UNAIDS, recommends that the shortage of health workers can be addressed by the following innovative approaches: task-shifting, creation of new cadres, changing role of nurses and involvement of PLWHA as ‘expert patients’ [20].
